# The Perceptions of Women with Gynecological Cancer after Radiotherapy Treatment: A Gender-Based Qualitative Study

**DOI:** 10.3390/healthcare11111580

**Published:** 2023-05-28

**Authors:** Alicia Uceda-Escobar, María Dolores Guerra-Martín, Alicia Botello-Hermosa

**Affiliations:** 1 Virgen Macarena University Hospital, 41009 Seville, Spain; alicia.uceda.sspa@juntadeandalucia.es; 2 Institute of Biomedicine of Seville (IBiS), 41013 Seville, Spain; 3 Faculty of Nursing, Physiotherapy and Podiatry, University of Seville, 41009 Seville, Spain; abotello@us.es

**Keywords:** attitude, gender, genital neoplasms, female, qualitative research, radiotherapy

## Abstract

Gynecological cancer is on the rise and radiotherapy is resorted to for its treatment, which affects the patients. This study was conducted following qualitative methodology to analyze women’s gender-based perceptions. The data were collected by means of semi-structured interviews. Five categories were defined: 1. feelings; 2. daily living activities; 3. role in the couple/family; 4. coping; and 5. knowledge and uncertainties. There was one emerging category: embarrassment and effects of toxicity. The qualitative data analysis was performed in Nudist NVivo V.11. It was concluded that the patients presented both positive and negative feelings, there were limitations to their daily living activities, their role in the couple/family was affected, they faced problems with resignation, emotional avoidance, and spirituality, they mostly stated having incomplete information, and they underwent uncomfortable situations due to the secondary effects of radiotherapy.

## 1. Introduction

Cancer is still one of the main causes of morbidity and mortality [1]. The number of cancer cases diagnosed in Spain has increased due to the population growth in recent years. According to the REDECAN calculations, an incidence of 280,100 cases in Spain was estimated in 2022 [1]. In turn, in the USA, this incidence was 1,918,030 cases in 2022 [2].

Gynecological cancer has also been affected by this increase; this type of cancer develops in the female reproductive organs (cervix, ovaries, uterus, vagina, vulva, and breasts) [3]. In the USA, the estimates for 2022 were 14,100 and 65,950 new cervical and uterine cancer cases, respectively [2]. In Spain, 2480 and 6773 new cervical and uterine cases were estimated [1].

Chemotherapy, radiotherapy, or both techniques are resorted to for cancer treatment [3]. The cervix (cervical) and uterus (endometrial) are the most frequent sites treated with radiotherapy. Both pathologies present with different staging according to the Federation of Gynecology and Obstetrics (FIGO) Classification [4], and the action protocol will depend on this.

Radiotherapy produces a number of adverse effects caused by the toxicity of the treatment, which are detectable in the treated and adjacent organs [4]. Some of these adverse effects or toxicities are as follows: 1. discomfort in the rectum; 2. lower limb lymphedema; 3. a risk of urinary incontinence and vaginal prolapse; 4. vaginal fibrosis [5]; 5. psychological effects (mood disorders, stress, and anxiety) [6]; and 6. radiodermatitis (cutaneous lesions produced by radiotherapy dose accumulation) [5,7,8].

During the clinical practice of radiotherapy nursing consultations, it has been observed that women who initiate treatment for gynecological cancer present feelings, uncertainties, and perceptions that they express in an active way, accompanied by non-verbal language. This is not merely a change in the physical health of the cancer patient, but a change in her feelings [9]. The feelings or sensations produced are normal and appear during the oncological process due to the situation experienced [10], a feeling inherent to “being ill” [11]. Some of these feelings are fear, anxiety, and wishes for the future, among others [9].

A person’s anxiety (a feeling of anguish that usually accompanies many diseases and does not allow the patients to feel at ease [12]) and fear or apprehension about something happening to them that is contrary to their wishes [13] generally emerge out of a lack of control over the situation they are experiencing, whether they will continue to live or not or if their routines will be interrupted, and due to medical terms of which they are unaware [9].

Due to its secondary effects, radiotherapy produces a change in the instrumental daily living activities of the women undergoing its treatment, that is, the activities related to independent life, which include cooking, shopping, house chores, working, and leisure, among others [14,15,16]. This is specifically due to vesico-rectal preparation (related to the large amount of water they have to drink and the administration of an enema prior to the radiotherapy session) and the dietary recommendations that should be followed during treatment [14,15]. This change comes when a woman assumes her disease, which has been explained from different points of view: Bayés [17] highlighted the stress generated and its characteristics, Holland [18] emphasized the possible influencing factors of this, whereas Kübler-Ross drew a similarity with the mourning phases [19].

Coping with this disease is linked to health personnel’s attitudes towards cancer. Peláez et al. [20] stated the importance of undergoing the disease process with psychological monitoring to assist with coping. Coping depends on several stressors [21], with different types of coping: 1. coping using resignation [22,23]; 2. coping using emotional avoidance [24]; and 3. coping using spirituality [24].

According to Gala et al., health personnel’s attitudes, i.e., not wanting to mention death or the disease and not looking at a patient’s face, can generate an uncomfortable situation for the patient, precluding the opportunity for her to freely express her opinions and instead inducing her to repress her feelings [22] Information sharing about the treatment process and the resulting patient uncertainty will impact the disease-coping strategies used [25,26,27].

According to the diverse scientific evidence about the oncological process, some needs emerge [28], and identifying them helps in providing a comprehensive service to patients [29]. Active listening plays an important role in detecting these needs and the disease process [30]. The patient’s wishes and desires for the future [31] are related to how they live with the disease [32].

On the other hand, it is convenient to note that gynecological cancer is associated with many gender-based stereotypes and roles [29] related to “being a woman”, which can influence the disease process. The uterus is culturally associated with motherhood, femininity, and sexuality [33]. In this sense, diverse scientific evidence asserts that cancer diagnoses constitute a stressful experience that supposes an enormous burden of anguish and distress, psychological problems, fear, and shame, among others [20,33], to which motherhood and its relationship with fertility should be added, as well as a woman’s own sexuality and role in the family/couple, which can be affected by such a diagnosis [11,23,34]. For this reason, it is also considered necessary to apply a gender-based perspective in this research to analyze the influence of these social and cultural factors on the experiences related to gynecological cancer.

The study objective was to analyze the perceptions of women with gynecological cancer after radiotherapy treatment from a gender-based perspective.

## 2. Material and Method

### 2.1. Research Design

The study was conducted following a qualitative methodology. An approach based on hermeneutic phenomenology was adopted [35], which was ideal for understanding the participants’ discourse, experiences, feelings, and fears, as well as for evaluating the quality of the nursing care received. This qualitative methodology was the closest to social reality and its complexity, constituting a rigorous and systematic method; in addition, it allowed for understanding the complex world of life experiences from the point of view of the people who underwent them, as they were explored in relation to their context and natural environment [35,36]. In addition, the holistic, dynamic, and individual dimensions were taken into account [37]. The study followed the Standards for Reporting Qualitative Research (SRQR).

### 2.2. Participants, Sampling and Recruitment

The women selected were those who had been diagnosed with gynecological cancer and were undergoing radiotherapy during the research period (from October 2021 to September 2022). The observation unit was the Radiotherapy and Radiophysics Oncology unit of a hospital in southern Spain. The participants were recruited through nursing consultations.

The segmentation criteria when selecting the sample were the following: 1. participants’ gender: female; 2. diagnosis: the sample consisted of women with gynecological cancer, focusing on cervical and endometrial cancer, who had undergone and concluded their radiotherapy treatment between May and September 2022; 3. coadjuvant chemotherapy or possible subsequent brachytherapy treatments were not taken into account; and 4 women treated by the hospital’s Radiotherapy and Radiophysics Oncology service. On the other hand, motherhood, nationality, and marital status were established as variability criteria.

### 2.3. Data Collection

For the data collection, semi-structured interviews were considered as the crucial method for answering the questions set out in the study objectives. In order to conduct these interviews, an ad hoc script was prepared with semi-structured questions and six analysis categories: 1. feelings; 2. daily living activities; 3. role in the couple/family; 4. coping; and 5. knowledge and uncertainties, which are presented in the following section.

The interviews lasted a mean of 20 min. For the discourse analysis, the interviews were transcribed after their audios were collected, always favoring the subjects’ privacy. Each participant was assigned a pseudonym, consisting of the letter “P” (patient) and a number (the order in which the interview was conducted), with “I” for the Interviewer. The data analysis was performed after the verbatim transcriptions. The qualitative data (words and texts) analysis consisted of a discourse analysis: separation by codes, code families, and categories, in order to understand the diversity of ideas gathered during the data collection. All of the above was carried out in the Nudist Nvivo software, version 11; this program was used as computational support in the analysis of the data of a qualitative nature (interview transcriptions, field diaries, and observation records, etc.) [38].

## 3. Results

### 3.1. Description of the Participants

The profiles of the participants interviewed are shown in Table 1, according to the segmentation and variability criteria.

### 3.2. Analysis Categories

The analysis categories are shown in Table 2, along with the frequency of their occurrence in the statements.

#### 3.2.1. Feelings

When the participants were asked “How did you react when you were informed about the need for radiotherapy treatment?”, we obtained answers from the patients with positive and negative feelings.

In relation to positive feelings, the patients considered the treatment as an improvement in the process that was necessary for a full recovery. Some verbatim excerpts are presented below:

*“Well, absolutely fine, because what I wanted was to get well…”*

[P6]

*“I really deal with this fine.”*

[P4]

*“I feel somehow calm. I mean, everything’s OK. In my country…I would’ve been operated on and just go home. …Here they treat me as they should and they follow me up by telephone and with the scanner. …I’m in good hands.”*

[P2]

Regarding the negative feelings when faced with the radiotherapy news, we found the following:

*“Pretty average…. As if they were telling YOU.”*

[P3]

*“In terms of mood…. I’m not OK.”*

[P5]

*“Bad, I felt really bad.”*

[P2]

Faced with the radiotherapy treatment news, some women stated being afraid, but others did not. As for those who felt fear:

*“Fear and sorrow.”*

[P2]

*“Yes, I was afraid.”*

[P5]

*“…now I’m concerned about dying…”*

[P8]

Regarding the women who were not afraid, we present the following verbatim excerpts:

*“I wasn’t afraid, as I’d already undergone chemotherapy, it was not that there was going to be anything puncturing me or that my hair would fall.”*

[P1]

*“Of course not, of course not. It’s kind of that I don’t believe it… I don’t believe what is happening to my sister. But I had it operated on on time…I’m living it kind of relaxed.”*

[P4]

Although not all of them did, some women expressed feelings of anxiety produced by the news about the need to undergo radiotherapy for their treatment. Regarding those that felt anxiety:

*“Yes, quite a lot. Really bad at the beginning. It’s only sometimes now.”*

[P8]

*“Yes, and I got very upset.”*

[P1]

As already commented, other women did not feel anxiety, as exemplified below:

*“Well… I think no. I believe that I’ve never been anxious, but never in my life, I think that not during this process either.”*

[P7]

*“Nothing, I’m pretty fearful, but I saw everything perfect.”*

[P6]

Another feeling expressed by the women was their wish to be healthy and finish the oncological process:

*“Ah, how much I want for all of this to end and get better. And be able to live in peace, I’ll have my reviews, but getting better, because now I’m almost always a wreck.”*

[P7]

*“I want to go out again, practice some sport, lead a normal life.”*

[P2]

#### 3.2.2. Instrumental Activities of Daily Living (IADLs)

When asked “How has radiotherapy treatment influenced your daily life?”, many of the study participants indicated some limitation or change in their IADLs after the radiotherapy treatment, as can be seen below in the following examples:

*“I used to work, I’m on leave now. I went out a whole lot more before and we traveled...”*

[P7]

*“Very limited, my life pace has changed”*

[P4]

*“I do almost the same things, but I’m very tired. Well, not the same things.”*

[P6]

*“Not the same, same things; because it hurts me down there.”*

[P9]

#### 3.2.3. Role in the Couple/Family

When asked “How has radiotherapy treatment affected your relationship with your couple/family?”, in the participants’ discourse, it was observed that the patients themselves were concerned about the well-being of their family members (husband or children, etc.), sometimes putting aside their own feelings and health status. We could see the stereotype of women in charge of the house chores and children, emphasizing the patients’ most maternal aspects.

*“It’s harder to tell your children… really hard… because when you’re a mother you think one way, but differently when you’re a daughter. And when you’re both you understand these things… One of them is 50 years old and the other is 48, no kids at all, but you go tell them that their mother, who is the one that’s going to live forever, the one to always be there… Well, what I have is cancer. For me, this word didn’t come out… but I hinted it to them and… that it was very hard for me to tell my children. Those around can see that I… that I can’t… That I don’t go shopping… when I lay down in bed all day, they’ve never seen this before.”*

[P3]

We also obtained a testimony that expressed certain concerns about future motherhood or fertility, but which indicated that this aspect ceased to be important when life itself was at stake. The following patient prioritized being alive and healthy over having children in the future:

*“I don’t see it…I’ve always wanted to be a mother, having children…but with this, I don’t even know if after this… come on, sure thing that I won’t be able to, so, well, it seems that I won’t have biological children. I’ve dealt really bad with this, but now there’s nothing else I can do, so I’ll fight for my own health. Being alive is enough for me.”*

[P7]

In the following statement, we can see how radiotherapy affected the role played by this woman in her environment. It affected finding a partner in this case:

*“I live alone, well, I’ve had my partners, but now like this....who’s going to put up with me. My sister really keeps an eye on me. I haven’t had children and now...no way...”*

[P8]

Regarding sexuality, it was difficult for the patients to speak freely about this topic during the interviews, which hindered obtaining results in this scope. According to what was inferred from the participants’ statements, it was shown that was a part of each person’s intimacy and was deeply affected by gynecological radiotherapy. In the following statement, the patient referred to certain limitation in her life and intimate relations due to the discomfort caused by the effects of radiotherapy.

*“It has changed it altogether, there’s no… it’s a difficult situation…as most of the days I don’t feel like it because I’m not OK, it’s not as before for me either… well you already know… kind of no in the state I’m in.”*

[P7]

#### 3.2.4. Coping

When asked “How are you coping with this process of radiotherapy treatment?”, the participants responded that coping with cancer depended on many stressors. This is why the attitudes can be quite fluctuating, as reflected by the following testimony:

*“It overwhelms me at some moments. Sometimes I cry and others I laugh, like everyone else.”*

[P3]

Regarding the answers obtained from the interviewed patients, they experienced coping from different perspectives. These different points of view about coping among the patients with gynecological cancer are analyzed below.

- Coping using resignation.

*“Well look, it had to be me and that’s it, and I’m going to put it on and I’m going to get well, and that’s it. Not with optimism but with resignation, kind of saying ‘Come on, it’s fine’.”*

[P3]

- Coping using emotional avoidance.

*“I get up, do my things and don’t think.”*

[P6]

- Coping using spirituality.

*“Nothing, I’m pretty fearful, but I saw everything perfect, I believe it’s in God’s hands.”*

[P6]

However, as a general attitude, although the testimonies sometimes reflected a positive or hopeful aspect, most of the patients showed deficient coping with the disease at some moment during the interviews, given that it is a difficult process.

*“I haven’t thought about how I’ve dealt with this, but… I believe that not very bad. I mean, this is tough both for me and for those around me…. They’ve helped me a lot, my family has helped all the time. They’ve let me fall down little, but I did have big relapses sometimes, especially at the beginning. Perhaps I’m stronger now.”*

[P7]

#### 3.2.5. Knowledge and Uncertainties

When asked, “after having the radiotherapy procedure explained to them, did they have doubts or uncertainties derived from their lack of knowledge?”, the patients who answered that the information was clear and of an excellent quality agreed that they had no doubts or uncertainties during the process, which brought them tranquility. The patients were informed by the radiotherapy oncologist and nurse at the beginning of the radiotherapy process.

*“Very well informed about everything; in fact, when the belly thing started, a little bad, we didn’t even scare because I already knew. And everything went really well, everything.”*

[P1]

A few of the patients who answered that they received little information about the procedure were asked about their doubts. Others researched their doubts on the internet.

*“They told me that I had to do radio, as far as I can remember, they didn’t explain much to me.”*

[P6]

*“I always think, well, that if they hide something from me…. And I say, well, if you don’t tell me the truth. And also, if I feel something, I mean if it’s not normal, if anything hurts, to see if I gave something and they don’t want to tell me…. I search in the Internet. I like to search because I research a lot, but I see bad things.”*

[P2]

*“I search because it’s an addiction. I mean, they’ve explained everything perfectly to me.”*

[P9]

#### 3.2.6. Emerging Category: Embarrassment and Effects of Toxicity

These statements show us that the patients underwent certain uncomfortable situations, which were mostly caused by the adverse effects of the radiotherapy. The participants focused their answers on rectal discomforts, with alterations in their intestinal transit, asthenia, or tiredness:

*“I used to get up really willing to do my things. I’m going to do this in the morning and that other thing… that’s over now. No lunch, because I say ‘What if then the belly swells before getting there.’”*

[P1]

*“Of course that my life pace has changed because I don’t feel like going to the beach…”*

[P4]

*“Last week I only didn’t have diarrhea on Sunday, this week so far it’s been every day…”*

[P3]

*“It’s hurting me a little for the belly. No lunch, because I say ‘What if then the belly swells before getting there.’”*

[P1]

*“The worst thing is tiredness…”*

[P6]

## 4. Discussion

Regarding positive feelings, although radiotherapy does have adverse toxic effects, in general, the women participating in this study had positive reactions to the treatment news, as they considered it necessary for recovering their health. In this sense, the treatment generated joy, relief, and hope in the patients, possibly because the demands related to the treatment came to an end. This positive view about the disease helps to counteract the negative effects of stress, as stated in previous studies on the topic [9,24].

Referring to negative feelings, the study by Rodríguez Fernández [21] mentioned that patients may have a pessimistic attitude due to the secondary and evolutive effects throughout their different sessions.

As stated in the other publications consulted, fear is one of the most frequent reactions after a cancer diagnosis, sometimes out of a lack of knowledge and sometimes due to stories told, myths, or previous experiences [9], where fear of death is common [9,21].

Referring to an absence of fear, this can be motivated by previous experiences, coping, or spirituality, as has been stated in other studies [39,40].

It is common for patients diagnosed with cancer to be anxious, as shown in our study [41], although the patients themselves sometimes cannot identify this feeling due to a lack of knowledge about how to handle their own feelings, as has been described in different studies [11,24].

Regarding wishes for the future and the will to live, the priorities in the life of the patients interviewed were healthy and related to finishing the oncological process [21,32].

According to the National Cancer Institute [16], IADLs are affected by a cancer diagnosis. Specifically, vesico-rectal preparation and dietary recommendations affect patients’ lives and activities [14,15]. The participants indicated limitations in their IADLs due to tiredness, which exerted an influence on their life pace, leisure activities, and work, which is in line with other studies [14,15,16,21,42].

The “role In the couple/family” category was grounded in gender-based roles, stereotypes, and social mandates, as well as in how all of this influenced the life experiences, feelings, coping, and discourse of the patients diagnosed with gynecological cancer. The testimonies showed certain concerns about the family role, that is, the caregiver role, which were influenced by the patients’ fluctuating perceptions of their body, femininity, and fertility that were produced during coping with the disease, as asserted in the scientific evidence [24,43,44].

Regarding future motherhood, different studies have indicated that priorities during the oncological process are variable and have highlighted the importance attained by survival [21,45]; our study confirms this idea: the patients prioritized being alive and healthy over having children in the future.

According to Olivares Crespo [11], there is a relationship between gynecological cancer processes and reduced self-esteem, as well as with couple, sexuality, and fertility problems. This could be observed in the answers given in our study.

It should be noted that there is little scientific evidence addressing the topics of fertility and sexuality in patients subjected to this type of treatment. As sexuality in women is still a complex subject matter in need of being addressed, sometimes even considered as a taboo and full of mystery and shame, it is generally a topic that women only discuss with their closest family members or about which they simply do not speak openly [11].

According to Lazarus and Folkman, coping with this disease depends on the patients’ cognitive and behavioral efforts to handle the stress that results from the internal and/or external demands inherent to the situation they are experiencing [21]. This coping can be experienced in different ways. 1. Using resignation: the patients settle for, tolerate, and cope patiently with the disease, with the definite objective of achieving well-being. This attitude would be similar to the disease acceptance phase in comparison with mourning, as evidenced by Kübler-Ross [19] and reflected in our study. 2. Using emotional avoidance: this is characterized by the removal of feelings and concealing of emotions. This type of coping appeared in our study. According to Enríquez Villota [24], this type of coping results in a lower compliance with treatments or in their abandonment. 3. Using spirituality: the studies consulted show that, in the face of severe physical diseases, both patients and family members resort to spirituality or religiousness to cope with them [39,46]. It has also been noticed that, when dealt with using spirituality or religiousness, the disease helps the patients to lower their levels of embarrassment, hostility, anxiety, and social isolation [46]. Some of the patients interviewed resorted to spirituality as a coping strategy, showing a more hopeful point of view.

Referring to doubts, knowledge, or uncertainties about the process, most of the patients answered with not having any doubts. Jaman-Mewes and Rivera [45] asserted that almost 50% of their patients were fully informed; among them, the profile corresponded to women aged less than 58 years old with breast gynecological cancer. This was in line with our study (58.1 years old); in addition, they were the family caregivers and played a protective role, which is characteristic of patients that better incorporate the information they receive, according to Jaman-Mewes and Rivera [45].

On the other hand, in relation to the patients who answered as having doubts, there were factors limiting the proper assimilation of the information provided to them: 1. denial and/or forgetfulness due to the patient’s anxiety; 2. the family hiding the diagnosis from the patient; and 3. the physician’s comfort and desire to avoid any embarrassing situation by not providing data about the diagnosis and treatment [21,24]. In this sense, the study patients resorted to the internet as an information source and common practice, as was the case in the studies consulted [47,48].

The patients stated that they underwent certain uncomfortable situations due to the secondary effects of radiotherapy. These uncomfortable situations may have made them deal worse with the treatment, influencing their mood, willingness to lead a social life, or even their everyday routines, which was in line with other studies [21,49,50]. These studies have also highlighted that these uncomfortable situations are transitory, disappearing or improving after the treatment and generating relief in the patients [49,50].

One of the study limitations was the reduced number of participants in view of such a specific and complex topic. Another limitation that may have exerted an influence is that sexuality is a very intimate subject matter and might have been conditioned by the place where the interviews were conducted.

## 5. Conclusions

The current study shows the perceptions of women with gynecological cancer after radiotherapy treatment from a gender-based perspective. Regarding their feelings, the women attested to having both positive (relief and hope about the treatment coming to an end) and negative feelings (fear and anxiety). In relation to their daily living activities, they indicated having experienced limitations due to tiredness, which exerted influences both on their work and leisure time. In relation to their role in the couple/family, this was affected by the disease and the treatment, by their perception of their body, and by their fluctuating femininity and fertility levels. In turn, as for coping, the patients experienced it using resignation, using emotional avoidance, and using spirituality. Regarding knowledge and uncertainties, most of the patients indicated having the full information and resorted to the internet in the case of any doubts. In relation to embarrassment, this was due to the secondary effects of radiotherapy, which exerts influences on mood and the treatment process.

A comprehensive approach to women in radiotherapy services is recommended, considering not only physical aspects, but also social and psychological ones. In addition, it would be convenient to include education programs, for example, on sexuality, differentiating it from fertility.

For an additional study, it is proposed to interview not only women, but also men with cancer in the pelvic area and health professionals involved in the radiotherapy process from different hospitals, all from a gender perspective in health. In addition, it is proposed that focus groups are carried out for the enrichment of the qualitative research. Additionally, the convenience of carrying out a quantitative study was observed, in order to be able to collect and analyze more data on the topic of this study.

## Figures and Tables

**Table 1:** Profiles of the women interviewed.

Code	Age	Pathology	Motherhood	Marital Status	Nationality
P1	74	Endometrial cancer	2 sons	Married	Spanish
P2	49	Endometrial cancer	No	No partner	Moroccan
P3	76	Endometrial cancer	2 sons—1 daughter	Married	Spanish
P4	76	Endometrial cancer	2 sons	Widow	Spanish
P5	57	Cervical cancer	3 sons—1 daughter	Married	Spanish
P6	66	Cervical cancer	2 sons	Married	Spanish
P7	35	Cervical cancer	No	With a partner	Spanish
P8	40	Endometrial cancer	No	No partner	Spanish
P9	50	Endometrial cancer	1 daughter	Widow	Spanish

Source: the authors.

**Table 2:** Analysis categories and their frequency in the statements.

Category	Frequency
1. Feelings	30
2. Daily living activities	14
3. Role in the couple/family	10
4. Coping	16
5. Knowledge and uncertainties	11

Source: the authors.

## Data Availability

Not applicable.
